# Age at first marriage, age at first sex, family size preferences, contraception and change in fertility among women in Uganda: analysis of the 2006–2016 period

**DOI:** 10.1186/s12905-020-0881-4

**Published:** 2020-01-16

**Authors:** Paulino Ariho, Allen Kabagenyi

**Affiliations:** 1 Department of Sociology and Social Administration, Kyambogo University, P.O. Box, 1, Kyambogo, Uganda; 2 Department of Population Studies, School of Statistics & Planning, College of Business and Management Sciences, Makerere University, P. O. Box, 7062 Kampala, Uganda

**Keywords:** Change in fertility, Age at first marriage, Age at first sex, Family size preferences, Contraceptive use, Children ever-born, Decomposition, Sub-Saharan Africa, Uganda

## Abstract

**Background:**

Uganda’s fertility was almost unchanging until the year 2006 when some reductions became visible. Compared to age at first marriage and contraceptive use, age at sexual debut and family size preferences are rarely examined in studies of fertility decline. In this study, we analyzed the contribution of age at first marriage, age at first sex, family size preferences and contraceptive use to change in fertility in Uganda between 2006 and 2016.

**Methods:**

Using data from the 2006 and 2016 Uganda Demographic and Health Survey (UDHS), we applied a nonlinear multivariate decomposition technique to quantify the contribution of age at first marriage, age at first sex, family size preference and contraceptive use to the change in fertility observed during the 2006–2016 period.

**Results:**

The findings indicate that 37 and 63% of the change in fertility observed between 2006 and 2016 was respectively associated with changing characteristics and changing fertility behavior of the women. Changes in proportion of women by; age at first marriage, age at first sex, family size preferences and contraceptive use were respectively associated with 20.6, 10.5 and 8.4% and 8.2% of the change in fertility but only fertility behavior resulting from age at first sex was significantly related to the change in fertility with a contribution of 43.5%.

**Conclusions:**

The study quantified the contribution of age at first marriage, age at first sex, family size preferences and contraceptive use to the change in fertility observed between 2006 and 2016. We highlight that of the four factors, only age at sexual debut made a significant contribution on the two components of the decomposition. There is need to address the low age at first sex, accessibility, demand for family planning services and youth-friendly family planning services to young unmarried women such that they can achieve their desired fertility. The contribution of other factors such as education attainment by women and place of residence and their relationship with changes in fertility calls for addressing if further reduction in fertility is to be realised.

## Introduction

Many developing countries in Latin America and Asia experienced rapid reductions in fertility rates and this was termed a reproductive revolution [1]. By comparison, Africa is yet to experience this revolution as the onset of its fertility transition occurred about two decades later and at a slower pace than in non-African developing countries [2]. The fertility decline in Sub-Saharan Africa (SSA) is characterized by country and regional variations in both the onset and pace [3, 4]. During 1950–1955, all sub-regions of SSA had fertility rates of above 6 births per woman [4].

In spite of the variations in pace and onset, all regions of SSA have experienced reductions in fertility. For instance, during the 2010–2015 period, the fertility of Southern Africa region declined to 2.5 births per woman while that of the Eastern and Western Africa regions slowly declined to 4.9 and 5.5 births per woman respectively during the same period [4]. A country’s demographic transition is associated with social and cultural norms that strongly influence reproductive behavior [5]. Some of these norms are those regarding the onset of sexual intercourse and union, contraception as well as the ideal size of the family. Studies have documented the importance of reductions in fertility desires for fertility transition especially in high fertility countries such as those in Africa [4, 6, 7]. Indeed, one of the major factors responsible for the reproductive revolution in Latin America and Asia was a decline in the desired family size [1]. The decline of fertility preferences that accompanies development leads to a decline in actual fertility as this facilitates the adoption of birth control [8].

Like fertility levels in SSA, the fertility desires in the region are generally high compared to other sub-regions globally [9]. For instance, in the year 2011, SSA had an average desired family size of 5.1 children per woman. This was higher than the averages for North Africa and the Middle East (3.2), Asia (2.7) and Latin America (2.7) [10]. The nearly constant and high desired number of children in SSA is responsible for the region’s persistent high fertility [2, 11]. SSA has experienced modest decreases in desired family size with an average decline of just 0.13 children per woman. Due to the slow pace of decline, it will take more than a century for SSA to reach a desired family size of two children [10]. The relatively high fertility desires in SSA are rooted in traditional pronatalist practices that also partly explain the lower prevalence of contraception in the region [2, 12]. Although studies have pointed to a positive relationship between the number of desired children and fertility, the relationship is not uniform for SSA. Despite experiencing increases in the number of desired children, Niger and Chad showed a slight decline in their levels of fertility while Mozambique witnessed a decline in the desired number of children yet its fertility increased [12].

In the East African region, a change in an ideal number of children was reported to be one of the factors driving the region’s change in fertility [11]. In Kenya, a shift toward wanting more children was one of the main factors that explained the reversal of the country’s fertility decline during the period in which the country experienced a stall in fertility transition [13]. While comparing Ugandan and Ethiopian fertility, it was asserted that fertility decline in the two countries can be attained when women realize their own desired family size [14].

Despite being largely prohibited in African societies, premarital sexual activity happens [15]. In many parts of Africa, first births precede formal marriage and in some cases, proof of fecundity is an important pre-condition to formalizing marriage [16]. In East Africa, premarital pregnancy is sometimes a driver of marriage rather than vice versa [17]. In Rwanda and Uganda, studies have reported significant associations between age at sexual debut and lifetime fertility [18, 19].

Uganda has persistently had high fertility. High fertility refers to a total fertility rate (TFR) of 5.0 or higher [6]. The persistent high fertility is partly attributable to cultural and religious preferences for large families that limit contraceptive use [20]. Although religious and cultural values in Uganda prohibit premarital childbearing, demographic and health survey results have consistently indicated that many Ugandan women engage in sex before marriage which exposes them to the risk of pregnancy [21]. For some young people in Uganda, early pregnancy is a positive incentive for early marriage and some young women are said to pierce condoms during sexual intercourse so that they can get pregnant and thus compel their partners into marriage [20]. Premarital sex thus creates a favorable ground for early pregnancy, early marriage, and early childbearing which have known implications on fertility levels and public health.

Until the year 2006, Uganda had experienced almost unchanging fertility and this can among others be linked to the nearly constant mean ideal number of children among women [22]. Since the year 2006, the TFR for Uganda has shown indications of a faster decline. Uganda’s TFR declined from 6.7 children per woman in 2006 to 5.4 children per woman in 2016 [21]. Studies on Uganda’s fertility have explored the role of factors such as education, woman’s contraceptive behavior, marriage and contraceptive use on fertility levels [20, 23–26]. In this paper, we analyzed the changes in fertility preferences, age at first marriage, age at first sex and contraception among women between 2006 and 2016 and also assessed the extent to which these changes have contributed to the changing fertility in Uganda while quantifying the independent contribution of each to the observed change in fertility.

## Materials and methods

We used the 2006 and 2016 Demographic and Health Surveys (DHSs) conducted among females aged 15–49 years in Uganda to analyze the change in fertility that was observed in Uganda between 2006 and 2016. The DHSs are nationally representative cross-sectional surveys. In both the 2006 and 2016 surveys, women were asked about their birth histories and this provided information on the total number of children ever born (CEB) which we used as our measure of fertility in the decomposition of the change in fertility. CEB is a measure of cumulative fertility and includes the total number of live births that the woman had ever had at the time of the survey. The DHS data were formally requested from Measure DHS which subsequently authorized the use. This study adopted a multivariate decomposition analysis that quantifies changes observed over time into components attributable to changing characteristics and changing behaviors to determine the contribution of changing the age at first marriage, family size preferences, age at first sex and contraception to change in fertility observed between 2006 and 2016.

### Inclusion and exclusion criteria

Our inclusion and exclusion criteria were based on the DHS question about the sexual activity of women. In the DHS, women were asked: *“how old were you when you had sexual intercourse for the very first time?”*. With this question, women who report that they have never had sex are given a code “0”. Such women by natural means are not exposed to the risk of pregnancy and consequently childbearing. We thus only included women who had ever had sex and excluded those who declared that they had never had sex (virgins) as these are considered not to have natural exposure to pregnancy. This inclusion and exclusion criteria makes it possible for women who had ever had sex but did not declare so to be excluded. This possibility is enhanced by the fact that questions on sexual activity are sensitive especially among young unmarried women in cultural contexts that discourage premarital sexual activity. Young unmarried women may not declare that they are sexually active yet indeed they are. This poses challenges of disclosure of information related to sexual activities.

### Variables

The dependent variable used in the study was the number of children ever born (CEB) to a female respondent in the 2006 and 2016 surveys. CEB is a measure of the total number of children born to a woman up to the moment at which the data are collected [27]. CEB looks at all children that were born alive to the woman and excludes stillbirths. This measure was selected over TFR which is considered a superior measure of fertility. TFR is a measure of the number of children that a woman who starts giving birth at the age of 15 would have by the end of the reproductive span (age 49 years) if the age-specific fertility schedule remains unchanged. It is a synthetic measure that assumes constant birthrates over the lifespan based on a hypothetical cohort of women of reproductive age and that no one will leave the hypothetical cohort [28]. Unlike CEB which is a measure of actual cumulated fertility by the woman, TFR relies on current behavior (last 3 or 5 years) and thus children born prior are not considered. Furthermore, fertility decisions such as those to do with giving birth and using contraception may be made based on the number of children that women or couples have already had. The major independent variables for this analysis are; family size preferences, age at first marriage, age at first sex and contraceptive use. We also include education, type of place of residence as other factors that may contribute to the change in fertility. Education specifies the level of education attained by the woman at the time of the survey. We classified education into three categories; no education (women who reported not to have attained any level of education), primary and secondary education. Type of place of residence looked at whether the woman resided in an urban or a rural area while contraceptive use sought information on whether the woman was currently using any contraceptive method or not. Age at first marriage specifies the age at which the woman first entered into a union. However, to cater for women who were sexually active but not in a union, a category for “never married” was created.

In the surveys, women are asked, *“If you could go back to the time you did not have any children and could choose exactly the number of children to have in your whole life, how many would that be?”* This question helps generate data on the ideal family size (family size preferences) for the individual woman. There was a category of women who gave a non-numeric response to this question. Such responses include; “It’s up to God”, “As many as I can support”, “I don’t know”. Data on age at first sex was obtained based on the question, *“how old were you when you had sexual intercourse for the very first time?”* we categorized women who had ever had sex into three major categories; younger than 15 years, 15–19 years and 20+ years. This was to enable the grouping of a sexual debut into very early adolescence and late adolescence and then the post-adolescence. Details of the questions asked regarding birth histories are in the demographic and health survey reports.

### Data analysis

In our analysis, we first described the 2006 and 2016 sample of women by age, education level, place of residence, age at first marriage, age at first sex, ideal family size and contraceptive use. We used the Pearson chi-squared test to assess whether between 2006 and 2016, there was a significant change in the composition of women by these characteristics. Although the study used CEB as the dependent variable, the age-specific fertility rates (ASFR) and TFR of the women were computed to compare the estimated fertility levels by age at first marriage, ideal family size preferred, age at first sex and contraception status of the women using the tfr2 module [29] for the two survey years. The ASFRs by the age at first sex, age at first marriage, the ideal number of children and contraception status are presented as Figs. 3, 4, 5, 6. Additional file 1 describes the primary statistics and calculations that were used to generate the data. The data was first weighted using a weighting variable generated using the sample weight variable in the DHS data coded as v005. The weighting took into account the complex sample design used in the DHS.

Finally, the study used decomposition analysis to quantify the contribution of the selected factors to the variation in cumulated fertility of the women between the years 2006 and 2016 using CEB as the outcome variable. Decomposition analysis was selected because; although they do not establish causation, decomposition methods are a useful approach that identifies the main sources of change in an outcome [30]. Multivariate decomposition methods analyze changes or differences in outcome variables into components of change and assess their relative importance. These changes reflect population characteristics that may directly or indirectly influence outcomes [31]. A non-linear multivariate decomposition (mvdcmp) technique that deals with count outcomes such as the number of children was applied. The decomposition technique partitions change over time into components attributable to changing effects and changing composition [32]. Specifically, the mvdcmp analysis technique portioned the change in CEB observed between 2006 and 2016 into two components (that is, changing characteristics of women and variation in effects of the characteristics on CEB) in an overall decomposition and isolated the unique contribution of each characteristic to each of the two components in a detailed decomposition [32]. Because CEB is a count, a Poisson regression model was selected for the multivariate decomposition. In the decomposition model, changing characteristics refers to a part of the observed change in fertility that is associated with differences in the composition of the women age 15–49 years by selected characteristics whereas variation in effects of characteristics refers to the part of the change that is associated with differences in fertility behaviors that are a result the characteristics. These are reflected in differences in coefficients and this part is thus also known as the coefficients’ effects [32]. In this study’s context, the coefficient effects represent variations in the risk of childbearing that was observed between 2006 and 2016. The coefficient effects indicate changes in the risk of childbearing for the women of selected characteristics over time. The changing characteristics component is labeled “E” in eq. 1 and Tables 3 and 4 while the coefficient effects component is labeled as C. To obtain the overall contribution of a characteristic to the change in fertility, the percentages for the various categories of a given characteristic are added together. The summarized decomposition equation is as below

1 $$ \overline{Y_{2016}}-\overline{Y_{2006}}=E+C $$

Where; $\overline{Y_{2016}}-\overline{Y_{2006}}$ is the Mean difference in children ever born between the year 2016 and the year 2006. Component E indicates what the change in fertility would be if the women in the 2016 survey were given the distribution of covariates on the women in the 2006 survey while C shows the would-be fertility variation if the 2006 women experienced the 2016 childbearing rates associated with the independent variables.

We run two decomposition models. In the first model, place of residence and education level were included as control variables while in the second decomposition model, we analyzed the association of contraceptive use, age at first marriage, age at first sex and family size preferences with the observed change in fertility. This was done because of the differential association of the four factors with place of residence and education attainment by women. For example, contraceptive use has generally been reported to be higher among women in urban areas, higher education categories compared to their counterparts in rural areas and lower education categories. Also, age at first marriage is lower among women in rural areas compared to those in urban areas.

## Results

The results displayed in Table 1 indicate the composition of women by their age, education, place of residence, contraceptive use, age at first marriage, family size preferences, age at first sexual intercourse and also shows whether there was any statistical difference in the composition of women by the characteristics between 2006 and 2016. The weighted sample of women was 7281 and 15,799 for the 2006 and 2016 survey respectively. The results indicate that there was no statistical difference in the age composition of women between 2006 and 2016. On the other hand, there were significant differences in the composition by education, place of residence, contraceptive use, age at first marriage, family size preferences and age at first sexual intercourse.

**Table 1:** Distribution of women in 2006 and 2016

Characteristic	2006, *n* = 7281	2016, *n* = 15,799	*p*-value
Age			
15–19	11.4	12.3	0.5575
20–24	21.7	22.3	
25–29	19.2	19.0	
30–34	16.7	15.9	
35–39	12.9	12.7	
40–44	10.1	10.1	
45–49	8.0	7.6	
Education			
No education	22.2	11.0	0.001
Primary	58.6	57.2	
Secondary	19.2	31.8	
Residence			
Urban	16.5	26.4	0.001
Rural	83.5	73.6	
Contraceptive use			
No	77.1	64.6	0.001
Yes	22.9	35.4	
Age at first marriage			
Not married	10.7	13.2	0.001
Below 15	15.2	11.2	
15–19	56.4	49.1	
20+	17.8	26.5	
Family size preferences			
0–2	7.5	7.1	< 0.001
3–4	40.8	47.6	
5+	48.0	43.0	
Non-numeric	3.7	2.3	
Age at first sexual intercourse			
Below 15	20.1	19.0	< 0.001
15–19	55.0	69.6	
20+	24.9	11.5	

Whereas the proportion of women who reported having no education was 22% in 2006, it was 11% in 2016. Furthermore, the percentage of women who reported to have attained at least secondary education was 19% in 2006 and 32% in 2016. This shows that the 2016 sample of women had more educated women than the 2006 sample and this may have implications on the levels of fertility over the two years. Similarly, the findings indicate that the proportion of women who were residing in urban areas was 16.5% and 26.4 in 2006 and 2016 respectively.

Twenty-three percent of the women that were interviewed in the 2006 UDHS reported that they were using a contraceptive method at the time of the survey compared to the 35% who did so in 2016. This shows that there were more contraceptive users in the 2016 sample and thus any fertility differences may be linked to this observation. The table also shows notable differences in the age at first marriage especially for the women whose marriage occurred at ages younger than 15 years and more than 19 years (20+ years). The proportion of women who reported age at first marriage as younger than 15 in the 2016 survey was lower than that of 2006 while that of their counterparts in the category of 20+ was higher than that of 2006. Generally, the proportion of women who reported their age at first sex to be 15–19 in both 2006 and 2016 was higher than those whose sexual debut was below 15 years and at least 20 years. The proportion of women who reported their age at sex debut as 15–19 years was 55% in 2006 and 69.6% in 2016. Table 1 shows that the proportion of those that began having sex aged at least 20 years was 24.9% in 2006 and 11.5% in 2016. This may point to possible early exposure to pregnancy and childbearing.

Regarding fertility preferences, Table 1 shows that in 2006, about 3.8% of the women gave a non-numeric response (such as; “It’s up to God”, “As many as I can support”, “I don’t know”) about their ideal family size. This proportion of women reduced to 2.3% in 2016. The proportion of women whose preference was 0–2 children was 7.5 and 7.1% in 2006 and 2016 respectively. Table 1 also indicates that the proportion of women who preferred 3–4 children increased from 40.8% in 2006 to 47.6% in 2016 while that which preferred a family size of at least five children was 48% in 2006 and 43% in 2016. This shows a slight decline in the proportion of women that preferred at least five children.

### Changes in fertility

**Fig. 1:**
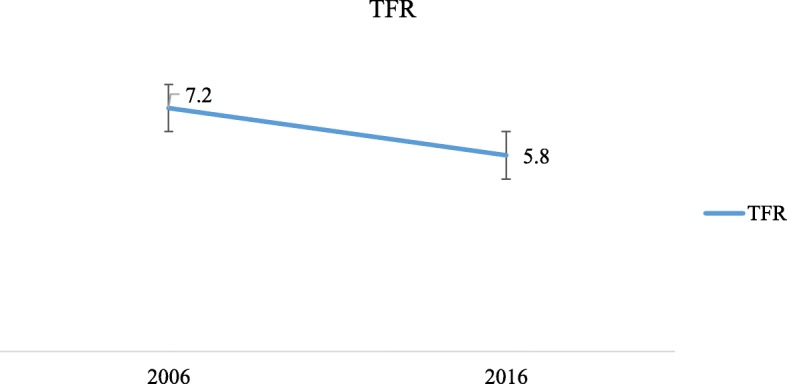
The 2006 and 2016 total fertility rate (TFR) estimated by STATA tfr2 tool

Changes in fertility in this study are described by changes in TFR and age-specific fertility rates (ASFR). The results in Fig. 1 show that the TFR reduced from 7.2 children per woman in 2006 to 5.8 children per woman in 2016.

**Fig. 2:**
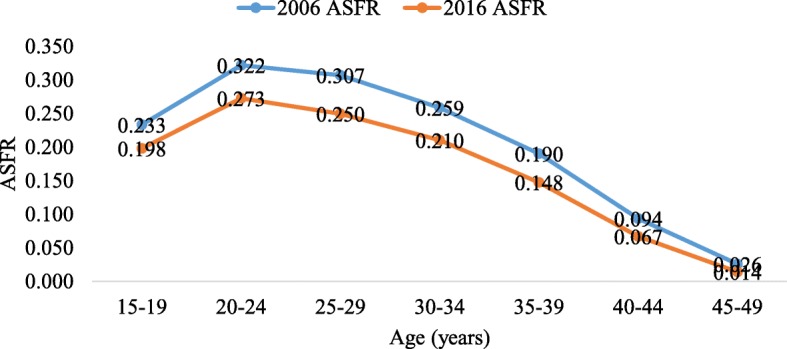
The 2006 and 2016 age-specific fertility rate (ASFR) estimated by STATA tfr2 tool

The findings in Fig. 2 show the ASFR for women in 2006 and 2016. The findings indicate that, generally, the 2006 ASFR was higher than that of 2016.

**Fig. 3:**
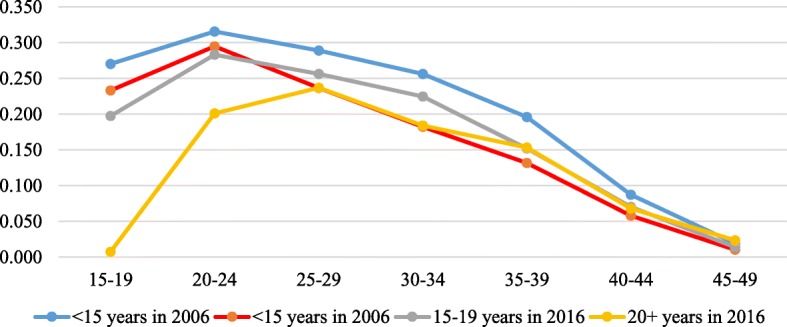
The age-specific fertility rate (ASFR) presented by the women’s reported age at first sex for the years 2006 and 2016. Rates were estimated using the STATA tfr2 module

**Fig. 4:**
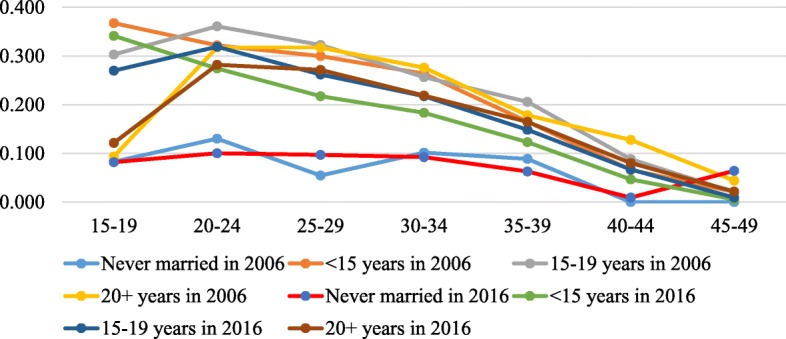
The age-specific fertility rate (ASFR) presented by the women’s reported age at first marriage for the years 2006 and 2016. Rates were estimated by the STATA tfr2 module

**Fig. 5:**
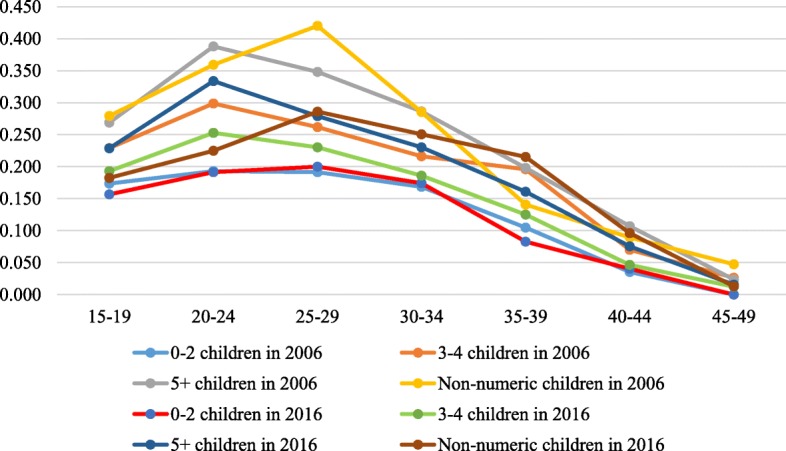
The age-specific fertility rate (ASFR) presented by the women’s reported ideal number of children for the years 2006 and 2016. Rates were estimated by the STATA tfr2 module

**Fig. 6:**
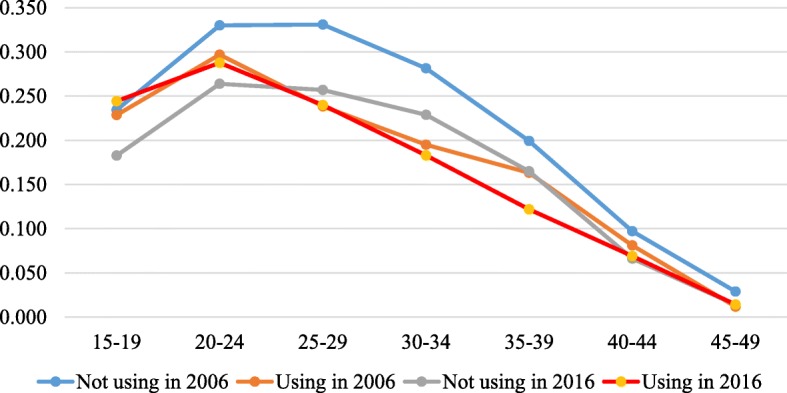
The age-specific fertility rate (ASFR) presented by the women’s contraceptive use for the years 2006 and 2016. Rates were estimated by the STATA tfr2 module

Figures 3, 4, 5, 6 in the appendix show the ASFRs by age at first sex, age at first marriage, ideal number of children and contraceptive use. In Table 2, we present the TFR by age at first sex, age at first marriage, ideal family size preferred by women and contraception status for the two survey years. The findings indicate that the TFR of women whose age at first sex was younger than 15 years declined from 7.2 in 2006 to 5.7 in 2016 whereas that of those whose age at first sex was 15–19 years, 20+ years reduced from 7.1 to 6.0 and 7.4 to 4.4 children per woman between the two survey years. Also, the findings show that the TFR slightly increased from 2.3 in 2006 to 2.5 children per woman in 2016 for women who were never married but reduced from 7.5 to 6.0, 7.8 to 6.5 and 6.8 to 5.8 for their counterparts whose age at first marriage was younger than 15 years, 15–19 years and 20+ years respectively between 2006 and 2016.

**Table 2:** TFR by age at first sex, age at first marriage, ideal family size and contraception status

Characteristic	2006				2016	
	TFR	*P* value	95% CI	TFR	*P* value	95% CI
Age at first sex						
< 15 years	7.2	< 0.001	6.692–7.640	5.7	< 0.001	5.458–6.012
15–19 years	7.1	< 0.001	6.813–7.380	6.0	< 0.001	5.836–6.139
20+	7.4	< 0.001	7.034–7.852	4.4	< 0.001	4.057–4.683
Age at first marriage						
Never married	2.3	< 0.001	1.685–2.889	2.5	< 0.001	1.956–3.121
< 15 years	7.5	< 0.001	6.975–8.078	6.0	< 0.001	5.580–6.344
15–19 years	7.8	< 0.001	7.505–8.066	6.5	< 0.001	6.286–6.647
20+	6.8	< 0.001	6.274–7.276	5.8	< 0.001	5.519–6.091
Ideal family size preferences						
0–2 children	4.3	< 0.001	3.710–4.957	4.2	< 0.001	3.812–4.643
3–4 children	6.5	< 0.001	6.161–6.824	5.2	< 0.001	5.054–5.412
5+ children	8.1	< 0.001	7.785–8.420	6.6	< 0.001	6.416–6.823
Non-numeric	8.1	< 0.001	6.933–9.291	6.3	< 0.001	5.354–7.338
Contraception status						
No	7.5	< 0.001	7.274–7.750	5.9	< 0.001	5.733–6.044
Yes	6.1	< 0.001	5.678–6.471	5.8	< 0.001	5.587–6.012

The findings in Table 2 show that TFR by ideal family size preferred was generally low for women in 2016 compared to their counterparts in 2006. The TFR of women who reported their ideal number of children as 0–2 children reduced 4.3 children per woman in 2006 to 4.2 children per woman in 2016. The TFR of women whose preferred number of children was 3–4 declined from 6.5 in 2006 to 5.2 children per woman in 2016 while that of their counterparts whose preferred number was at least five (5+) declined from 8.1 to 6.6 and those who gave a numerical response to the question on ideal family size reduced from 8.1 to 6.3 children per woman. Also, the results indicate that the TFR for women who were currently using family planning methods slightly reduced from 6.1 children per woman in 2006 to 5.8 in 2016 whereas that of their counterparts who belonged to the category of “not using” family planning methods reduced from 7.5 to 5.9. The findings thus reveal that whereas there was an estimated difference in TFR of 1.4 children per woman in 2006, this difference was only 0.1 in 2016. This may in part be due to the relatively large reduction in the TFR of women that were not currently using contraceptive methods compared to that experienced by women who were using contraceptives.

### Decomposition of change in fertility

The decomposition results indicate that the observed change in fertility between 2006 and 2016 among the women who had ever had sex can be attributed to both the changes in characteristics of women and changes in fertility behavior. The results in Table 3 show that changes in characteristics of women contributed 37.1% of the change in fertility while the remaining 62.9% of the change was due to the change in the effects of characteristics on the fertility behavior of the women. The 37.1% in Table 3 is obtained as the ratio of the coefficient on E to the coefficient on R (− 6.2923 divided by − 16.956) while 62.9% is − 10.663 divided by − 16.956.

**Table 3:** Overall Decomposition results

Component	Coefficient	*P* value	95% CI	Percent.
E	−6.2923	< 0.001	−7.30 -5.28	37.1
C	−10.663	< 0.001	−12.91 -8.41	62.9
R	−16.956	< 0.001	−18.94 -14.97	100.0

E = component explaining changing characteristics/endowments of women.

C=Component explaining coefficient effects.

R = Sum of E and C.

In Table 4, the results show that the observed change in fertility is associated with changes in; education level of women, place of residence, contraceptive use, age at first marriage, ideal family size preferences and age at sexual debut. However, of age at first marriage, ideal family size preferences and age at first sexual intercourse, only the change in age at sexual debut had a significant contribution to the component of the decomposition that is attributable to changing fertility behavior.

**Table 4:** Detailed decomposition of changes in fertility for the study period

E					C			
Variable	Coef	*P*-value	95% CI	%	Coef	*P*-value	95% CI	%
Education								
No education	1.00							
Primary	0.16	< 0.001	0.13–0.19	*−0.9*	−6.26	< 0.001	−9.37 -3.16	*36.9*
Secondary	−3.91	< 0.001	−4.39 -3.43	*23.1*	−3.19	< 0.001	− 4.83 -1.56	*18.8*
Residence								
Urban	1.00							
Rural	−0.89	< 0.001	−1.09 -0.69	*5.2*	−8.06	0.006	− 13.75-2.36	*47.5*
Contraceptive use								
No	1.00							
Yes	1.40	< 0.001	1.22–1.57	*−8.2*	0.19	0.712	−0.80- 1.17	−1.1
Age at first marriage								
Not married	1.00							
Below 15	−4.35	< 0.001	−4.74 -3.95	*25.6*	−2.77	0.169	−6.72 1.18	16.3
15–19	− 7.49	< 0.001	−8.18-6.80	*44.2*	−10.68	0.144	−24.99 -3.63	63.0
20+	8.35	< 0.001	7.61 9.08	*−49.2*	−1.21	0.569	−5.38-2.95	7.1
Family size preferences								
0–2	1.00							
3–4	0.43	0.002	0.16 0.70	*−2.5*	−3.59	0.174	−8.76 1.58	21.2
5+	−1.37	< 0.001	−1.60 -1.15	*8.1*	−2.05	0.501	−8.04 3.93	12.1
Non-numeric	−0.47	< 0.001	−0.55 -0.39	*2.8*	0.38	0.196	−0.02 0.97	−2.3
Age at first sexual intercourse								
Below 15	1.00							
15–19	−1.13	< 0.001	−1.41 -0.86	*6.7*	−3.14	0.053	−6.33 0.44	18.5
20+	2.92	< 0.001	2.61 3.24	*−17.2*	−7.37	< 0.001	−9.7 -5.00	*43.5*
Constant					37.10	0.021	5.61 68.59	− 218.8

Coef = coefficient × 1000, CI = Confidence Interval.

For each factor, the percentage contributions of the categories are added to obtain the overall contribution of the factor to the change in fertility. Our analysis indicates that overall, education accounted for 22.2% of the observed change in fertility. Specifically, the difference in fertility would increase by 23.1% if the proportion of women who had attained at least a secondary level of education in 2016 was the same as that of 2006. Relatedly, a change in the proportion of women who were residing in rural areas contributed 5.2% to the observed change in fertility while the observed increase in contraceptive use was responsible for 8.2% of the reduction in fertility.

Table 4 findings indicate that age at first marriage (20.6%), family size preference (8.4%), age at sexual debut (− 10.5%) and contraceptive use (− 8.2%). The negative percentage indicates the expected increase in the fertility difference if there was no change in age at first sex. Regarding the effects of the characteristics, our findings in Table 4 indicate that the fertility behavior resulting from education, place of residence and age at first sex significantly contributed to the 2006–2016 reduction in fertility. In terms of percentage, leaving other factors constant, the behavioral component resulting from changes in educational attainment accounted for 55.8% of the change. Similarly, the place of residence contributed 47.5% to the observed change in fertility that is associated with changing fertility behavior while age at first sex accounted for an overall percentage of 43.5%. When education is dropped from the model, age at first sex is shown to account for a combined percentage of 85.5% of the change attributed to the behavioral changes whereas place of residence contributes 39.8% to the change in fertility.

Furthermore, we conducted the decomposition analysis using a model that did not include education level and place of residence and the results showed that the percent contribution associated with the C component was insignificant while that associated with the C component increased to 96.9%. Furthermore, the detailed decomposition results revealed that only age at first sex made a significant contribution to the change in fertility associated with the E component of the decomposition. This shows that the contribution associated with changes in contraception, age at first marriage and family size preferences may be linked to the place of residence and the education level attained by the women and that age at sexual debut is a key factor associated with the fertility preferences. The results are presented in Tables 5 and 6.

**Table 5:** Overall Decomposition results (education level and place of residence excluded from the model)

Component	Coef	Std. Err.	P-value	95% CI	Percent
E	−0.52	0.464	0.259	1.43–0.39	3.1
C	−16.43	1.135	< 0.001	− 18.66--14.21	96.9
Overall	−16.96	1.0375	*< 0.001*	−18.99--14.92	100.0

**Table 6:** Detailed decomposition of changes in fertility for the study period (education level and place of residence excluded from the model)

Variable	Coef	*P*-value	95% CI	%	Coef	*P*-value	95% CI	%
Contraceptive use								
No	1.00				1.00			
Yes	0.23	0.180	−0.10-0.56	−1.3	0.76	0.137	0.24–1.75	−4.5
Age at first marriage								
Not married	1.00				1.00			
Below 15	−1.02	0.196	−2.55-0.52	6.0	−2.79	0.150	−6.60-1.01	16.5
15–19	−1.74	0.195	−4.38-0.89	10.3	−11.29	0.109	−25.12-2.53	66.6
20+	1.88	0.192	−0.95-4.70	−11.1	− 1.55	0.467	−5.73-2.63	9.2
Family size preferences								
0–2	1.00				1.00			
3–4	0.13	0.217	0.07–0.33	−0.7	−3.93	0.146	−9.22-1.37	23.2
5+	−0.37	0.196	−0.94-0.19	2.2	−1.32	0.672	−7.41–4.77	7.8
Non-numeric	−0.13	0.197	0.33–0.07	0.8	0.48	0.109	−0.11-1.06	−2.8
Age at first sexual intercourse								
Below 15	1.00				1.00			
15–19	−0.33	0.196	− 0.82-0.17	1.9	−4.97	0.003	−8.21--1.74	29.3
20+	0.82	0.164	0.34–1.98	−4.9	−10.15	< 0.001	−12.49--7.81	59.9
Constant					18.34	0.18	−8.21–44.90	− 108.2

## Discussion

In this paper, we find that changes in fertility behavior over the 2006–2016 period accounted for the biggest share in contributing to the observed change in fertility. When education, place of residence were dropped from the model, the fertility behavior component associated with age at first sex increased in terms of percentage contribution to the change in fertility whereas the same component associated with age at first marriage and ideal family size remained insignificant at the 5% level of significance.

Our findings indicate that over the period the sexual behavior of the women especially those aged 20+ years accounted for 43.5% of the observed change in fertility attributed to changing fertility behavior. Continued shifts in the age at entry into sexual intercourse will play a very significant role in Uganda’s demographic transition. These results are important for high fertility countries that are in the initial stages of the demographic transition [4, 9, 25]. Delayed sexual debut implies delayed exposure to the risk of pregnancy and childbearing and this influences the fertility performance of a woman. While traditional societal norms in Uganda prohibit sexual activity and pregnancy before marriage, many young people engage in premarital sex [20], Our findings are consistent with those from studies conducted in some other developing countries which have found fertility decline to have been influenced by delayed sexual debut. Studies in Namibia [33] and Rwanda [18] have found age at first sex to significantly influence fertility levels. In the Rwandan study, low fertility was associated with a delayed sexual debut.

Our findings also highlight the importance of age at first marriage in determining the levels and trends of fertility. Our findings may be explained by the observation that between 2006 and 2016, there was an increase in the proportion of women who reported the age at first marriage 20+ and a reduction in those that reported age at first marriage as below 15. This shows that there was a rise in age at which the Ugandan women first entered union or marriage. Rising age at first marriage is very crucial for the attainment of significant declines on fertility as long as frequent sexual exposure and childbearing are restricted to within marriage. Age at first marriage and its importance in the onset of fertility transition has been studied by Hertrich (2017) who contends that fertility transition is highly unlikely where women enter first unions at very early ages [34]. The results by Hetrich [34] revealed that for a large number of countries in SSA, a change in age at first marriage was more of a precursor of the initiation of fertility decline than a component of fertility transition. Age at first marriage is known to be influenced by education. In Uganda’s context, a continued rise in age at first union may be facilitated by attainment of at least a secondary level of education by women. Our findings point to possible faster declines in fertility as the majority of women in Uganda attain at least a secondary level of education. With the continued implementation of Universal secondary education by the government of Uganda, efforts should be made to minimize rates of dropout from school by women.

We find that family size preferences played a key role in the observed changes in fertility. We show that fertility preferences in the form of the ideal number of children preferred contributed significantly to Uganda’s observed change in fertility between 2006 and 2016. This paper echoes what previous studies have asserted about the importance of family size preferences in early fertility transitions. A desire for large families in African countries accounted for high fertility in SSA and therefore SSA’s transition to replacement level fertility cannot proceed unless large declines in desired family size occur [11, 31]. Relatedly, the pronatalist nature of African societies as reflected in preferences for larger family sizes partly explains the slow and weaker fertility decline African countries [2, 31]. For high fertility countries, studies have reported that fertility desires are more important for fertility reductions compared to contraceptive use [6] and fertility decline can be achieved when there is a large proportion of population desiring smaller family sizes [7] and having reduced mean age at childbearing [35]. Our findings also concur with the observation that high fertility in the early stages of the demographic transition is the consequence of high desired family size [25, 32]. Our findings imply that continued change in attitudes towards large families is paramount for sustainable declines in fertility in Uganda. The diffusion of information about methods of birth control is an important mechanism of fertility change [2].

In agreement with studies [3, 11, 13, 36–39] conducted elsewhere, our findings indicate that although the proportion of the women who were currently using contraceptives increased by 11.5% between 2006 and 2016, this was associated with 8.2% of the decline in fertility observed between 2006 and 2016. This finding highlights that contraceptive use by Ugandan women will be very key to Uganda’s fertility transition. On the other hand, this paper partly disagrees with the findings of [40] which reported a weak effect of contraceptive use in explaining Nepal’s fertility decline.

The strength of this manuscript is that the analysis is based on nationally representative survey data. Demographic and health surveys are among the reliable sources of data for the study of levels and trends of fertility and other demographic indicators in developing countries. Furthermore, the analysis technique used facilitates the portioning of change in an outcome over time into components attributable to changing characteristics of women and changing reproductive behavior. The multivariate decomposition also partitions the two components into portions that represent the unique contribution of each characteristic to each of the two components. Although most demographers and other researchers on fertility consider marriage as the exposure to childbearing, pregnancy, and childbearing may begin before marriage. Excluding these women who have out of wedlock births thus creates a vacuum on the reproductive health experiences of such women.

Our analysis technique is limited to analyzing differences between two groups only. It was thus not possible for us to conduct a decomposition analysis that includes more than two survey years. We pooled the 2006 and 2016 datasets to conduct a decomposition analysis. Demographic transitions are known to take longer periods and thus may require data over long intervals to provide a detailed explanation of such transitions. The 2006–2016 period presents a decade in which Uganda’s fertility has undergone some visible changes and is thus suitable for the analysis of changes in fertility. This enables us not only to observe changes in reproductive behaviors but also shifts in the socio-demographic composition of the population. The period 2006–2016 was also characterized by major policy and program changes that may be associated with demographic changes in Uganda. During this period, Uganda’s population policy (first promulgated in 1995) was revised in 2008. The revised policy highlighted the importance of providing reproductive health services to address persistent high fertility in Uganda [41]. This may have come with some effects on fertility in Uganda. However, this study did not analyse the effect of the policy. Similarly, education, a widely reported factor that influences fertility declines, for example in urban Uganda [23] also underwent significant policy changes. The 2006–2016 period is associated with a change in the education policy in Uganda as the country became one of the first countries in Sub-Saharan Africa to introduce universal secondary education in 2007 [42]. This Policy 2007 was introduced by the government of Uganda as part of the implementation of the Poverty Eradication Action Plan 2005–2010 (PEAP) and this can partly be linked to the increase in the population with tertiary education from 3% in 2002 to 4.3% in 2014 [43]. We are unable to directly assess the contribution of the universalization of education as a policy to the observed change in fertility.

Furthermore, because we used cross-sectional surveys we did not determine the cause-effect relationships but rather quantified the contribution of the factors (age at first sex, age at first marriage, family size preference and contraceptive use) associated with the change in fertility observed between 2006 and 2016. The study’s inclusion criteria were based on the question on the sexual activity of women. This question tends to be sensitive in settings where unmarried people are expected to abstain from sex until they are married. The possibility of either underreporting and/or misreporting on this question is thus increased. This could have excluded some women who had ever had sex but did not declare. Because premarital childbearing is largely frowned upon in most African societies, women are more likely to report that at the time of their first sex, they were married [17]. This may lead to the underestimation of premarital and early childbearing. Another limitation is that whereas DHS are generally a good source of data for the analysis of fertility, the fertility data are prone to recall issues and possible backdating of births as women try to avoid completing the birth history questions [17]. We also note that it has been reported that estimates of fertility based on births in the last three years often lead to an underestimation in most of the surveys with poor quality fertility data from retrospective birth histories [4]. Furthermore, DHS data on fertility may not be of sufficient quality to examine trends in fertility especially when using two data points [44]. However, our study is not aimed at examining trends but rather to quantify the factors contributing to the difference in fertility levels between the years 2006 and 2016 using decomposition analysis.

## Conclusions

The fertility of the Ugandan women who had ever had sex reduced from 7.2 children in 2006 to 5.8 children in 2016. We have assessed whether age at first marriage, family size preferences, age at first sex and contraceptive use have contributed to this observed change in fertility. The decomposition helped to determine the independent contribution of age at first marriage, family size preferences and age at first sex to the change in fertility observed between 2006 and 2016. We highlight that with continued reductions in family size preferences, there is a need to address the accessibility of family planning services such that women can achieve their desired fertility. There is also a need for increased effort to have a conducive family planning environment to reach more people with messages about services and the associated benefits. The study points to the fact that age at first sex has a bigger effect on fertility changes. With the age at first sexual debut for women generally lower than the age at their first marriage, the need for friendly family planning services especially for young unmarried people is paramount. Policies and programs need to be strengthened to tackle the effect of age at sexual debut on fertility behavior and its implications for achieving a fertility transition.

## Supplementary information

**Additional file 1.** The primary statistics and calculations used to generate the data.

## Data Availability

The datasets used for this study are publicly available through the link https://dhsprogram.com/data/available-datasets.cfm.

## Abbreviations

ANOVA: Analysis of variance
CEB: Number of children ever born
CI: Confidence interval
SSA: Sub Saharan Africa
UDHS: Uganda demographic and health survey
