# Supplementary material for: Age at first marriage, age at first sex, family size preferences, contraception and change in fertility among women in Uganda: analysis of the 2006–2016 period
Source: BMC Womens Health. 2020 Jan 16;20:8. doi: 10.1186/s12905-020-0881-4 (PMC6966849; doi:10.1186/s12905-020-0881-4)
Supplement: Supplementary file 1 — Additional file 1. The primary statistics and calculations used to generate the data. [file 12905_2020_881_MOESM1_ESM.docx]

**The primary statistics and calculations used to generate the data.**

The age specific fertility rate (ASFR) and total fertility rate (TFR) are two popular measures of fertility which are estimated basing on births to women aged 15-49 years in the three years preceding the survey.

ASFR is obtained as the ratio of births that occur to women of a particular age group to the total number of women in the age group; ASFR= $\frac{Births to women in age group}{women in the age group}$. This sometimes multiplied by a constant such as 100 or 1000

TFR is calculated as the summation of ASFR multiplied by women. TFR=$5\times\sum ASFR$. TFR assumes a hypothetical cohort of women and that the ASFR will remain constant and can thus be said to be said to be a hypothetical period measure of fertility.

CEB is a cumulative measure of fertility that is obtained by counting the total number of live births that a woman has had up to the time of the survey. The responses can be zero (for women who have never given birth) or a number as reported by the woman. This indicates the actual fertility of the woman up to the time of the survey.

Poisson regression model is used in regressions that deal with count outcomes. In the context of our study, since CEB is a count, the Poisson regression was used. This by default outputs coefficients, standard errors, z-values, p values and the 95% confidence interval of the estimates. The coefficients are however pose challenges of interpretation and are thus exponentiated to yield incident rate ratios (IRR). The IRR indicate both the direction and magnitude of the risk of childbearing for a particular category relative to the reference category of a variable. IRR values that are greater than 1 indicate increased relative risk while those less than 1 indicated reduced relative risk. Due to the fact that CEB varies with age as older women are most likely to have a higher CEB compared to young women at the time of the survey, the Poisson regression allows age to use as an offset variable.

$\ln\left( \mu_{i} \right)= \alpha+X_{i}\beta_{i}$+$\ln(age)$

Where, $\mu_{i}$is the expected number of children born to a respondent based on the respondent’s demographic and socioeconomic characteristics; $X_{i}$ are independent variables; $\alpha$ is a constant and $\beta_{i}$ represents coefficients associated with the independent variables. $\ln\left( age \right),$ is the offset variable and age is the current age of the woman

The nonlinear decomposition (mvdcmp) model also outputs percentage contributions in addition to the statistical outputs of regression parameters. The percentages indicate the unique percentage contribution of each selected category of a variable to the differences/changes in an aggregate outcome.

$\overline{Y_{B}}-\overline{Y_{A}}=\overline{F\left( X_{A}\beta_{A} \right)}-\overline{F\left( X_{B}\beta_{B} \right)}$ **(**a)

The equation 3.2a can be further decomposed to equation 3.2b below

$\overline{Y_{B}}-\overline{Y_{A}}=\left\{ \overline{F\left( X_{A}\beta_{A} \right)}-\overline{F\left( X_{B}\beta_{A} \right)} \right\}+\left\{ \overline{F\left( X_{B}\beta_{A} \right)}-\overline{F\left( X_{B}\beta_{B} \right)} \right\}$ **(**b)

The summarized form of equation 3.2b is as in equation 3.2c

$\overline{Y_{B}}-\overline{Y_{A}}= E + C$ (c)

Where; $\overline{Y_{B}}$ is the mean number of children ever born in 2016 and $\overline{Y_{A}}$ is the mean number of children ever born in 2006, $\overline{Y_{B}}-\overline{Y_{A}}$ is the Mean difference in children ever born between Year B and year A, *F*(*·*) is a logarithm function mapping a linear combination of *X* (*Xβ*) to *Y,* X represents predictors
